# Minimization of Surface Roughness and Temperature during Turning of Aluminum 6061 Using Response Surface Methodology and Desirability Function Analysis

**DOI:** 10.3390/ma15217638

**Published:** 2022-10-30

**Authors:** Endalkachew Mosisa Gutema, Mahesh Gopal, Hirpa G. Lemu

**Affiliations:** 1 Department of Mechanical Engineering, College of Engineering and Technology, Wollega University, Nekemte P.O. Box 395, Ethiopia; 2 Faculty of Science and Technology, University of Stavanger, N-4036 Stavanger, Norway

**Keywords:** cutting speed, aluminum 6061, response surface methodology, rate of feed, ANOVA, cutting depth, desirability function, tool nose radius

## Abstract

Aluminum alloy is the second most abundant metal on Earth, known for its wide range of utilization in commercial goods due to its heat capacity and tensile strength. This study examines the effect of nose radius on the turning process. Further, it explores the implications of cutting parameters such as the cutting speed, the rate of feed, the cutting depth, and the nose radius of the tool. The trials were carried out with an Al 6061 workpiece and an Al_2_O_3_-coated carbide tool as the cutter, utilizing the response surface methodology. A mathematical model was developed to investigate the performance characteristics of the turning operation using the analysis of variance method. The multi-response desirability function analysis combines individual desirability values to create a composite desirability value. The ideal parameter levels were determined using the composite desirability value, and the significant influence of parameters was assessed. The obtained optimum surface roughness and temperature parameters are at a cutting speed of 116.37 m/min, a rate of feed of 0.408 mm/rev, a cutting depth of 0.538 mm, and a tool nose radius of 0.20 mm. The related ideal surface roughness and temperature values are 0.374 µm and 27.439 °C. The optimal overall desirability value is 0.829, close to the target response.

## 1. Introduction

The ability to produce lightweight materials has created the path for the automotive and aerospace industries to reduce, for instance, the overall weight of vehicles [1]. To achieve this weight reduction, aluminum materials are used in various engineering fields to replace iron and steel, due to their superior mechanical properties including a high stiffness-to-weight ratio, physical and thermal qualities, corrosion resistance, and recyclability [2]. Aluminum and its alloys, on the other hand, have poor machinability. As a result, extensive research into studying performance attributes and efficient machining technologies are underway to lower the total costs.

To assess flank wear of the cutting tool in machining [3], experiments were conducted to investigate the cutting performance of aluminum alloy LM 25, which was reinforced with green-bonded silicon carbide. The study employed the response surface methodology (RSM) and desirability function analysis (DFA) to improve the surface roughness. A drilling process in aluminum-silicon carbide was performed [4] using the analysis of variance (ANOVA) and Taguchi methodologies to increase material removal rate (MRR) and to decrease surface roughness, where the RSM was employed to assess the tool’s surface waviness, thrust force, burr height, and wear. Rajmohan and Palanikumar [5] used Al 356 aluminum bonded with silicon carbide composition in the drilling experiment. In the study reported in [6], the composite desirability (CD) value of numerous performance attributes, such as surface quality and cutting force, was transformed into a single performance feature. Taguchi and DFA were used to optimize the parameters of aluminum-silicon carbide. The research work in [7] reported that the RSM and DFA enhanced the machining settings to maximize tool life and minimize energy usage during the Al 7075 alloy machining. The study used ANOVA to investigate the aluminum alloy, while the RSM was utilized to develop the mathematical model. The cutting speed was the most critical parameter that influenced the experiment compared to other parameters; further, sensitivity analysis was carried out to study the cutting force [8]. Mugendiran et al. [9] improved an aluminum alloy’s surface quality and wall thickness using the RSM and ANOVA. The RSM and the genetic algorithm were employed for the experimentation to optimize the aluminum sample. The input parameters considered were rotation speed, the rate of feed, the axial cutting and radial cutting depth, and tool rake angle [10]. Furthermore, the author in [11] optimized surface quality using the RSM and DFA of aluminum alloys. The RSM and DFA were also used to maximize aluminum alloy energy utilization, surface waviness, and material removal rate (MRR) by taking the cutting depth, the rate of feed, and the cutting speed into account [1,12,13].

The RSM is frequently used in conjunction with desirability functions to forecast more optimum results [14,15,16,17]. The research reported in [18] constructed an empirical model to anticipate overcut in machining using the Box Behnken design and compared the empirical model with the RSM model. The model corresponds well with the experimental data, and the model was compared with the RSM. DFA is a prominent method used in industry and academia to optimize several responses [19]. The author in [20] introduced the RSM and DFA as an optimizing tool to select the best option in turning replies into desirability indices ranging from 0 to 1. The response of predetermined maximum or minimum values was identified with process parameters inside a given range. The response was then transformed into the desired value via one-sided transformation in the desirability-based method. A numerical optimization method and desirability analysis were used to optimize the wear rate [21]. The author [22] employed the Taguchi method to investigate and determine the influencing variables using ANOVA. DFA was used as the performance index of the output by computing the composite DFA values. The research reported in [23] carried out three optimizations: (1) quality optimization, (2) economic optimization and (3) combined optimization, using ANOVA, the RSM, and DFA to minimize surface unevenness and wear in the tool. The desirability function combined with the Taguchi approach allows optimization of the multi-response concerns such as surface irregularity and MRR [24], optimization of the coating breadth, thickness, and interface temperature [25] and optimizing machining pressure and surface roughness [26]. An experimental study was conducted by [27] to optimize the microhardness and surface quality of IN625 material using Taguchi L9 optimization and Super Ranking techniques. The input parameters were laser power, scan speed, and hatch distance. The authors in [28] used the ANOVA, the RSM, fuzzy MCDM, fuzzy AHP, and fuzzy TOPSIS to optimize titanium (Ti6Al4V) alloy using a electrical wire discharge machining process to optimize the cutting speed, MRR, and surface roughness. The result emphasizes that the RSM is an effective tool for the design of experiments. The author [29] experimented by considering layer thickness, building orientation, raster angle, raster width, and air gap as input parameters and the output parameter as impact strength, flexural strength and tensile strength during processing by using fused-filament fabrication methodology in AM technology. The naked mole-rat algorithm (NMRA) was applied to solve the optimization process. The ball end milling operation was performed by [30] using hardened 55NiCrMoV6 steel material considering the cutting speed and surface inclination angle as input parameter to optimize the cutting force by using the RSM. The results indicated that the surface inclination angle has a significant influencing parameter compared to others. Aluminum 6061 is a precipitation-hardened alloy with excellent properties such as high ultimate tensile strength and yield strength that are desirable for many applications. It also contains significant elements of magnesium and silicon. Therefore, careful consideration should be taken during the machining process. The literature indicates that the desirability function technique is a proper method to enhance the multi-response optimization processes, such as those experienced in machining aluminum and its alloys [31,32].

Utilizing ANOVA and RSM, a lot of research have been reported on aluminum 6061 as the workpiece material. However, the application of ANOVA, the RSM, and DFA to optimize the input and output parameters during the turning of aluminum 6061 is not well documented in the literature. DFA application, in particular, has significant benefits to optimize and reduce several criteria simultaneously and to bring out the process changes that lead to better product quality and higher productivity.

In this study, experiments were conducted using the design of experiment (DoE) strategy and optimized using the RSM. The DoE is necessary to study the relationship between the multiple input and output variables and gain knowledge to estimate the best-operating conditions. Furthermore, the DoE was used to determine the individual and interactive effects of variables influencing the output result during measurements. In addition, DFA was used to convert the multiple response characteristics into single response characteristics and calculate the ideal machining environment of the process parameters to reduce surface roughness and temperature.

## 2. Methods, Materials and Process Parameters

### 2.1. Materials and Experimentation Setup

In this experiment, untreated aluminum 6061 with a diameter of 50 mm and a length of 100 mm was used as the working material. Table 1 shows the chemical properties of aluminum 6161 material.

**Figure 1:**
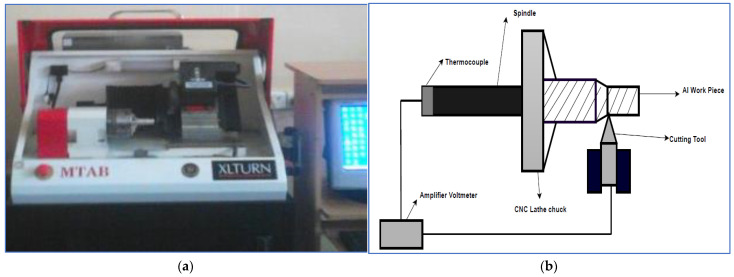
Experimental setup.

The trials were performed in dry conditions on an XLTURN-CNC lathe (MTAB, Tamilnadu, India), Figure 1a, with an Al_2_O_3_-coated carbide tool by using a Sandvik Coromant T-Max P Turning Tool Holder (Sandviken, Sweden). The machining parameters considered were the cutting speed, the rate of feed, the cutting depth, and the nose radius of the tool. A 1 mm hole was bored into the aluminum material, and the sample was positioned 10 mm beneath the cutting surface. As illustrated in Figure 1b, a K-type thermocouple (Shanghai MKYD Instrument, Shanghai, China) was used to detect the temperature, and a SURFTEST SJ-201 surface roughness tester (Mitutoyo America Corporation, Aurora, IL, USA) was used to assess the roughness of the surface.

### 2.2. Process Parameter Identification for Experiment

For the experimental investigation, the following process factors have an impact on surface roughness and temperature were identified as per the recommendations of machining conditions and input parameters in [33]. The output responses were formulated as follows.

- Minimization of the surface roughness (Ra in μm); the cutting factor equation is:

Minimize: Ra = c (V_c_^k^_1_ F_z_ ^k^_2_ D_c_ ^k^_3_ R_n_ ^k^_4_) (1)

- Minimization of the temperature (Temp, °C) using the cutting parameters

Minimize: Temp = c (V_c_^k^_1_ F_z_ ^k^_2_ D_c_ ^k^_3_ R_n_ ^k^_4_) (2)

where k_1_, k_2_, k_3_, and k_4_ are the model parameters (estimated from experimental data) and c is the response error.

### 2.3. The Creation of a Design Matrix and the Selection of Parameter Levels

The DoE was used in the creation of a design matrix, selection of parameter levels and the experiments, while the operating limits of all evaluation criteria were used to determine the levels of parameters. As the DoE is a systematic approach used to solve engineering problems, its application reduces the number of experiments and gives a high level of control. The operating ranges of all parameters were determined through trial runs, with one parameter modified while the others remained fixed. Trial runs before the stated parameters were used to identify the upper-bound (+2) and lower-bound (2) levels of all five independent variables, resulting in the precise predicted values shown in Table 2 and Table 3. The intermediate levels of all variables, 1, 0, and +1, were calculated via interpolation [34]. The design matrix chosen to conduct the experiments using the DoE method was a four-factor central composite rotatable design (CCD) consisting of 30 sets of coded conditions and a full replication, where 24 are non-center points and 6 are center points.

### 2.4. Surface Roughness Prediction Using a Response Surface Model

The experimental results were examined methodically using the Design-Expert software V11 (StatEase, Minneapolis, MN, USA). A second-order quadratic model is developed to predict surface roughness. As reported in [35], ANOVA has been used to determine the model’s suitability. The ANOVA table for Ra prediction is shown in Table 4.

The F-value of 19.96 suggests that the model is significant, where this large F-value might occur due to noise of 0.01% of the time. *p*-values less than 0.0500 indicate that the model terms, i.e., the model terms V_c_, D_c_, V_c,_ F_z_, and V_c_^2^ are significant. On the other hand, values over 0.1000 suggest that the model terms are not significant. Model reduction may help for a model with many insignificant terms (except those necessary to enable hierarchy).

The F-value of 0.2783 for the lack of fit suggests that it is minimal compared to the pure error. A substantial lack of fit F-value may occur 95.96% of the time due to noise, and a non-significant lack of fit is preferred. The following Design-Expert software’s (StatEase, Minneapolis, MN, USA) regression equation of the objective factors was obtained upon running the regression.

Ra = +0.786250 − 0.006694 × V_c_ + 0.476852 × F_z_ + 0.089583 × D_c_ − 0.110417 × R_n_ − 0.007407 × V_c_ × F_z_ − 3.69886 × 10^−17^× V_c_ × D_c_ − 0.000417 × V_c_ × R_n_ v 0.208333 × F_z_ D_c_ + 0.277778 × F_z_ × R_n_ − 1.50104 × 10^−15^ × D_c_ R_n_ + 0.000033V_c_^2^ + 0.462963 × F_z_^2^ − 7.93479 × 10^−16^ D_c_^2^ + 0.062500 × R_n_^2^ (3)

### 2.5. Temperature Prediction Using a Response Surface Model

A second-order quadratic model was developed to predict temperature. ANOVA analysis was used to determine the model’s fitness. The ANOVA table for the temperature prediction is shown in Table 5.

The F-value of 786.09 for the model indicates that it is significant. An F-value this large may be attributable to noise merely 0.01 percent of the time. Significant model terms have *p*-values less than 0.0500. The following model terms are significant: V_c_, F_z_, D_c_, R_n_, V_c_ D_c_, V_c_ R_n_, F_z_ D_c_, F_z_ R_n_, D_c_ R_n_, V_c_^2^, F_z_^2^, D_c_^2^, and R_n_^2^. Values greater than 0.1000 indicate that the model terms are insignificant.

As can be observed from the table, the F-value for lack of fit is 0.2783, which indicates that the lack of fit is negligible compared to the pure error. A significant lack of fit F-value due to noise has a 96.06% chance of occurring, but a non-significant lack of fit was preferred. The regression equation for the Design-Expert software (StatEase, Minneapolis, MN, USA) in terms of actual variables is provided below.

Temp = +123.32917 − 1.86083 × V_c_ − 19.67593 × F_z_ + 29.35417 × D_c_ − 43.10417 × R_n_ − 0.023148 × V_c_ × F_z_ − 0.260417 × V_c_ × D_c_ + 0.431250 × V_c_ × R_n_ − 23.26389 × F_z_ × D_c_ + 23.95833 × F_z_ × R_n_ − 28.59375 × D_c_ × R_n_ + 0.009097 × V_c_^2^ + 44.36728 × F_z_^2^ + 14.60937 × D_c_^2^ + 7.42187 × R_n_^2^ (4)

## 3. Discussion of Results

### 3.1. Interaction Effect of Surface Roughness

**Figure 2:**
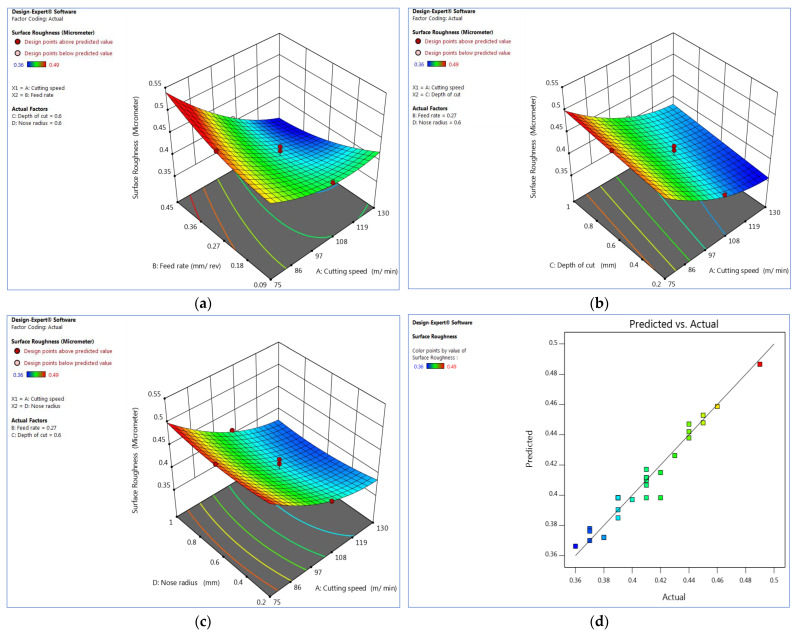
Interaction effect in terms of surface roughness of (**a**) V_c_ vs. F_z_, (**b**) V_c_ vs. D_c_, (**c**) V_c_ vs. R_n_ and (**d**) plot of actual vs. predicted values. Plots produced using Design-Expert software V11 (StatEase, Minneapolis, MN, USA).

Figure 2a depicts the interaction plot between the cutting speed (V_c_) and the rate of feed (Fz) in terms of surface roughness (µm). The cutting speed increases consistently, the surface waviness decreases, and the rate of feed increases; the surface roughness increases correspondingly. The cutting speed is between 90 and 105 m/min, the surface waviness value is low, and the rate of feed is low, at 0.09 mm/rev. The result shows that the surface waviness is negligible at a medium cutting speed and a low rate of feed. On the other hand, changes in the feed rate significantly impact both low and high cutting speeds. The cutting speed and the rate of feed determine surface finish and MRR. The ANOVA was also used to validate the result (Table 4).

The interaction impact of the cutting speed and the cutting depth on surface roughness is shown in Figure 2b. The cutting depth is low at a higher cutting speed substantially impacts surface roughness. The cutting depth is a critical cutting parameter that influences the whole process, including stability, cutting forces, vibrations, and spindle load. The surface roughness is lower at a higher cutting speed and a lower depth of cut. The effect of the cutting speed and tool nose radius on surface roughness is shown in Figure 2c. Lower cutting speeds result in higher surface roughness, while lower tool nose radius results in significantly lower surface roughness. The surface roughness significantly increases between the tool nose radius of 0.2 mm and 0.4 mm. At all cutting speeds, the same pattern emerges. The results revealed that the industry deserves a suitable surface roughness; the nose radius should be 0.8 mm and 1 mm.

Figure 2d depicts a graph of observed values vs. predicted values, and it assists in identifying the observations. The 45° line evenly divides the data points.

### 3.2. Interaction Effect of Temperature

**Figure 3:**
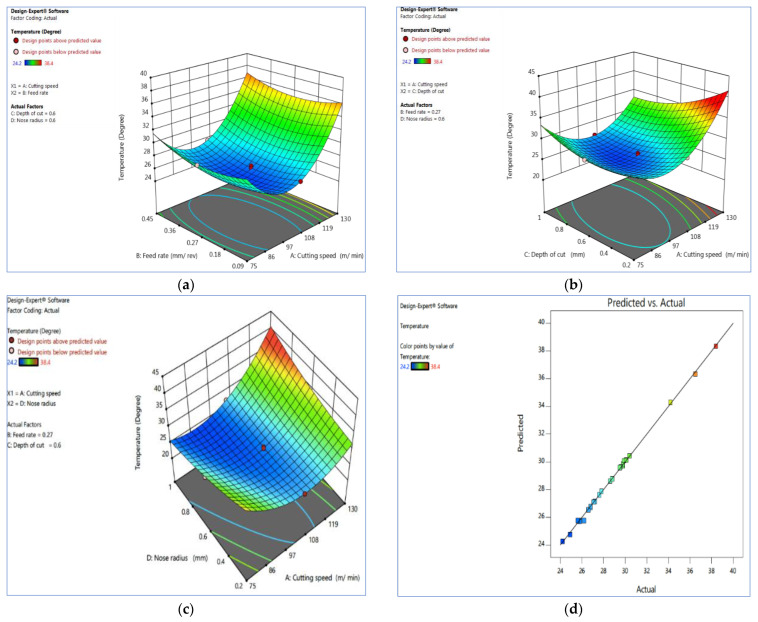
Interaction effect of temperature (**a**) V_c_ vs. F_z,_ (**b**) V_c_ vs. D_c_, (**c**) V_c_ vs. R_n_ and (**d**) actual vs. predicted plot. Plots produced using Design-Expert software V11 (StatEase, Minneapolis, MN, USA).

The temperature interaction plot between the cutting speed and the rate of feed is depicted in Figure 3a using the interaction effect. When the cutting speed is between 90 and 105 m/min, the temperature is relatively low, and it is also inadequate when the rate of feed is 0.27 to 0.36 mm/rev. At a medium cutting speed and rate of feed, the temperature is low. The ANOVA may also be used to validate the result (Table 5).

Figure 3b shows the effect of temperature on the cutting speed and the cutting depth. A smaller cutting depth significantly influences the temperature reduction. The cutting force increases when there is an increase in the cutting depth and the rate of feed, reducing with an increasing cutting speed.

Figure 3c demonstrates the effect of temperature interaction on the cutting speed and tool nose radius. The temperature rises dramatically with increasing the cutting speed and falls significantly with increasing tool nose radius, with the temperature decreasing noticeably between 0.6 and 1 mm.

The result indicates that to maintain a low temperature in the work piece, the nose radius should be between 0.8 and 1 mm. Figure 3d depicts a graph of observed vs. predicted values, which aids in detecting observations that the model fails to predict. The 45° line should split the data points evenly.

### 3.3. Desirability Function Approach

Researchers have employed various optimization approaches to increase product quality and productivity [7,31,32]. The studies indicate that a desirability function analysis improves the process parameters. The following procedures were used to optimize the input parameters using DFA and the RSM.

The work reported in [20] suggested the individual desirability index (di) for the comparable responses, where two desirability functions were considered based on the response characteristics, namely (a) the smaller, the better and (b) the larger, the better. The experiment was carried out to determine the features of electro-discharge machining (EDM) using an optimization-based desirability technique [36]. The experiment analysis was performed on hardened 55NiCrMoV6 steel to minimize cutting force and vibration. The signal-to-noise ratio (S/N) and grey relational analysis optimization techniques were performed, considering surface inclination angle and tool overhang as input parameters. According to the optimization results, changes in tool overhang increase the cutting force and vibration [37].

(a) The smaller the better: This method aims to reduce the output variable to the smallest possible value; hence, the smaller, the better characteristic is utilized to establish distinct desirability levels. To normalize their values and evaluate individual desirability indexes, the smaller, the better desirability function is utilized within the range of [0, 1]. When the response must be lowered, according to Harrington [38], Equation (5) defines the smaller, the better sort of quality characteristic.

The desirability functions di can be expressed as.

(5) $$\mathrm{di}=\left\{\begin{array}{c} 1\\ 0 \end{array}\;{\left(\frac{y_{m\;}-y_{\max}}{y_{\mathrm{tgt}}-{\;y}_{\max}}\right)}^{r}\;\right.$$

where y_m_ is minimized and

$$y_{m}\;<\;\;y_{\mathrm{tar}},\;{\;y}_{\mathrm{tar}}\;<\;y_{m\;}<\;y_{\max},\;r\;\geq \;0,\;\mathrm{and}\;y_{m}\;<\;{\;y}_{\max}$$

In this case, y_tar_ is the response’s lowest value y_m_, i.e., y_tar_ = y_min_.

Equation (5) can be modified as follows (Equation (6)).

(6) $$\mathrm{di}=\left\{\begin{array}{c} 1\\ 0 \end{array}\;{\left(\frac{y_{m\;}-y_{\max}}{y_{\min}-{\;y}_{\max}}\right)}^{r}\right.$$

where $y_{m}$ < $y_{\min}$, $y_{\min}$ < $y_{m}$< $y_{\max}$, r ≥ 0 and $y_{m}$ < $y_{\max}$

The condition is most attractive when y_m_ is smaller than y_min_ and the individual desire score is 1. When y_m_ deviates from y_min_, the value of d_i_ declines until it approaches “0” when y_i_ exceeds y_max_. In all other cases, the d_i_ values produced are in the range [0, 1]. The exponent w represents the weights allocated to each response depending on their relevance. Equation (7) computes the composite desirability as the geometric mean of each experimental circumstance’s desires.

(b) Composite Desirability (CD):

The composite desirability is expressed as:

(7) CD=D1W1× D2W2× D3 W3 × ……… 1N

where N is the number of responses, D_1_, D_2_, and D_3_ are individual desirability indexes, and W_1_, W_2_, and W_3_ and weight assigned responses, where w = $\overset{N}{\underset{i}{\sum }}W_{i\;}$= 1.

If any of the replies is entirely unacceptable, the value of CD becomes zero, i.e., Di = 0.

(c) Choosing the optimum parameter combination and level: A developed composite desirability value suggests that the product is of quality. As a result, the composite desirability is used to evaluate the parameter result and ideal level for each manageable parameter.

#### 3.3.1. Desirability Function Approach for Surface Roughness

The desirability functions presented in Equation (6) are used to calculate the individual desirability of surface unevenness. The minimum surface unevenness value is 0.32 µm, and the maximum value is 0.49 µm. Surface roughness increase is given equal the weightage (w_1_ = w_2_ = 1).

(8) $$d_{\mathrm{temp}}=\left\{\begin{array}{c} 1\\ 0 \end{array}\;{\left(\frac{y_{m\;}-0.49}{0.32-0.49}\right)}^{1}\right.$$

where $y_{m}$ < 0.32, 0.32 < $y_{m\;}$ < 0.49, r ≥ 0 and $y_{m}$ < 0.49.

Equation (9) was employed to compute the surface roughness based on the responses under consideration.

(9) CD=Dµmi 12  where i = 1 to 30

#### 3.3.2. Desirability Function Approach for Temperature

Individual desirability of temperature is calculated using the desirability functions as given in Equation (6). Surface roughness increases by giving equal weightage (w_1_ = w_2_ = 1), the minimum temperature value is 24.2°, and the maximum temperature value is 38.8°.

(10) $$d_{\mathrm{temp}}=\left\{\begin{array}{c} 1\\ 0 \end{array}\;{\left(\frac{y_{m}-38.8}{24.2-38.8}\right)}^{1}\right.$$

where $y_{m}$ < 24.2, 24.2 < $y_{m\;}$ < 38.8, r ≥ 0 and $y_{m}$ < 38.8.

Equation (9) computes the surface roughness based on the responses under consideration.

(11) CD=Dtemp112  where i = 1 to 30

#### 3.3.3. Process Parameter Multi-Response Optimization for Lowest Surface Imperfection and Temperature

Świercz et al. [39] used a multi-response optimization (MRO) technique for DFA. The optimization process was used, and the use of a statistical alternative to specifying the test to be performed earlier. Nowadays, the desirability function is extensively used to reduce multi-response situations into a single response [40]. The author [41] used the RSM and DFA to optimize the results. The highest overall desirability factor settings are found under the optimal parameter situations. The simultaneous goal function is a geometric mean of all updated replies. To minimize surface roughness and temperature, a multi-response optimization analysis of process parameters (Table 6) was performed using Design Expert V11 (StatEase, Minneapolis, MN, USA). Models were used to optimize surface roughness and temperature. The measure of the solution to fulfill the specified objectives for all responses are reviewed in MRO.

**Figure 4:**
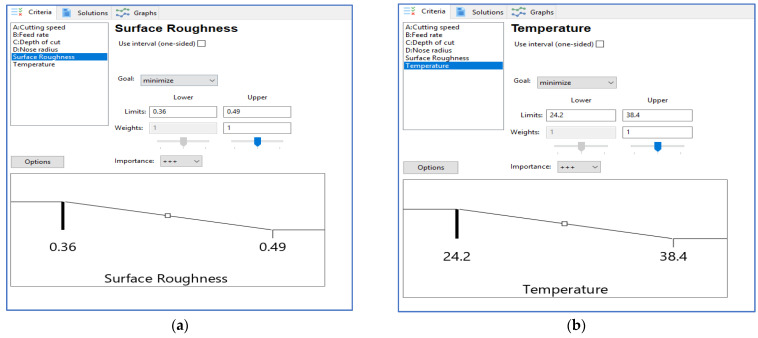
Criteria to (**a**) minimize µm and (**b**) minimize temp (T°).

The objectives of the two optimization approaches, as shown in Table 6 and Figure 4a,b, include criteria for reducing surface roughness and temperature, respectively. For instantaneous optimization, each response must have a low and high value given to each objective. The optimization is performed by a set of objectives, which have been applied to the variables and replies. The goal of the replies was to “minimize”. A weight can be used for a purpose to affect the shape of its desirability function. The results remain organized in order of preference, with the most desirable appearing first.

### 3.4. Desirability Function Analysis 

To achieve low surface roughness and temperature, desirability function analysis optimization was used. The two responses have the same goal, i.e., ‘minimize’ surface roughness and temperature. The depreciation seeks to get high-quality finished machined products with lower temperatures for more excellent performance and cost savings. These investigations targeted surface roughness and temperature characteristics are ‘smaller-is-better’. The desirability-based approach finds many best solutions, and the option with the most significant desirability is preferred: the best 63 solutions were acquired in the optimization.

Table 7 shows that only five higher desirability alternatives were chosen for the investigation, with 1 having tremendous desirability (D = 0.829). The optimal turning conditions are a cutting speed of 116.37 m/min, a rate of feed of 0.408 mm/rev, a cutting depth of 0.538 mm, and a tool nose radius of 0.20 mm. The ideal surface roughness and temperature are 0.374 µm and 27.439°. The smaller, the better principle was selected.

**Figure 5:**
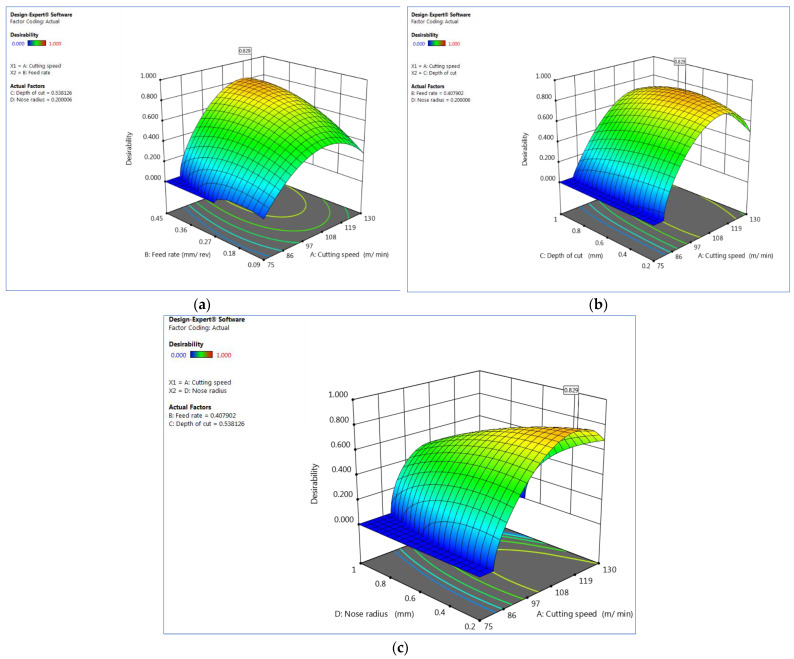
Estimated surface plots on desirability (**a**) V_c_ vs. F_z_, (**b**) V_c_ vs. D_c_ and (**c**) V_c_ vs. R_n_. Plots produced using Design-Expert software V11 (StatEase, Minneapolis, MN, USA).

**Figure 6:**
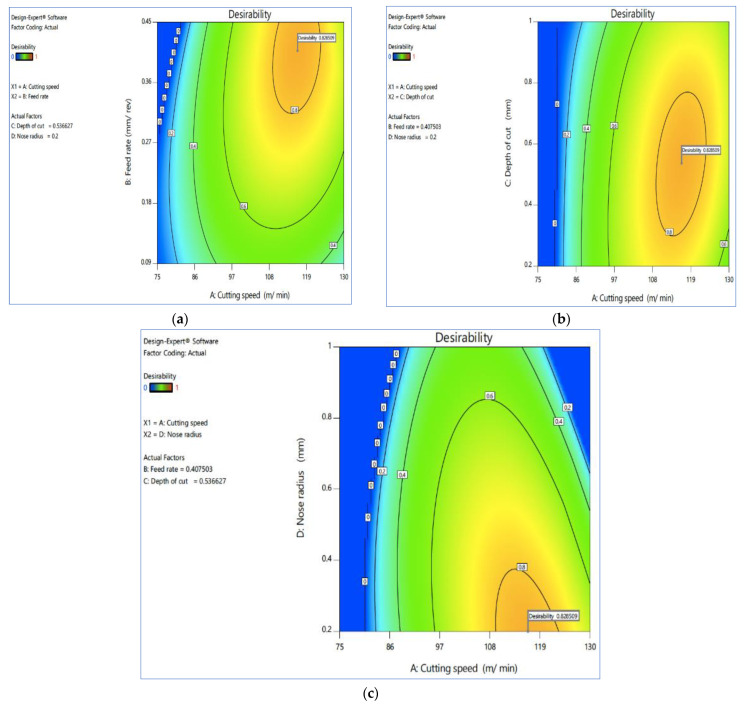
Contour plot on desirability for (**a**) V_c_ vs. F_z_, (**b**) V_c_ vs. D_c_ and (**c**) V_c_ vs. R_n_. Plots produced using Design-Expert software V11 (StatEase, Minneapolis, MN, USA).

The estimated surface over desirability for V_c_ vs. F_z_, V_c_ vs. D_c_ and V_c_ vs. R_n_ are shown in Figure 5a–c, while Figure 6a–c shown the contour plots on the desirability, respectively. The surface plots on the desirability, shown in Figure 5, are two-dimensional representations of a three-dimensional connection, where variables are given on the x- and y-axes and a smooth surface defining the dependent variables is shown on the z-axis. The contour plots on desirability are also shown in Figure 6a–c. A contour plot diagram is employed to investigate the relationship between the three variables. Two independent variables are displayed on the x-axis and y-axis, while one dependent variable is displayed on the z-axis in these plots. Contour plots aid in the identification of combinations that provide satisfactory results. These statistics also show that the best response was obtained using a higher cutting speed, a lower rate of feed, and a low cutting depth.

**Figure 7:**
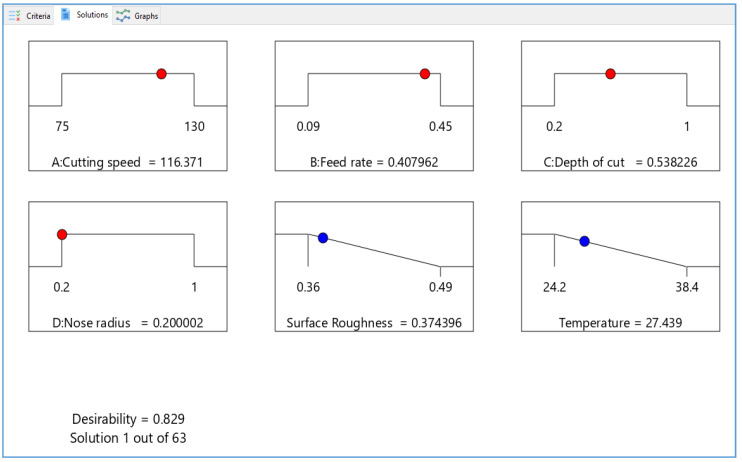
Optimal parameter ramps function graphs and combined optimization.

**Figure 8:**
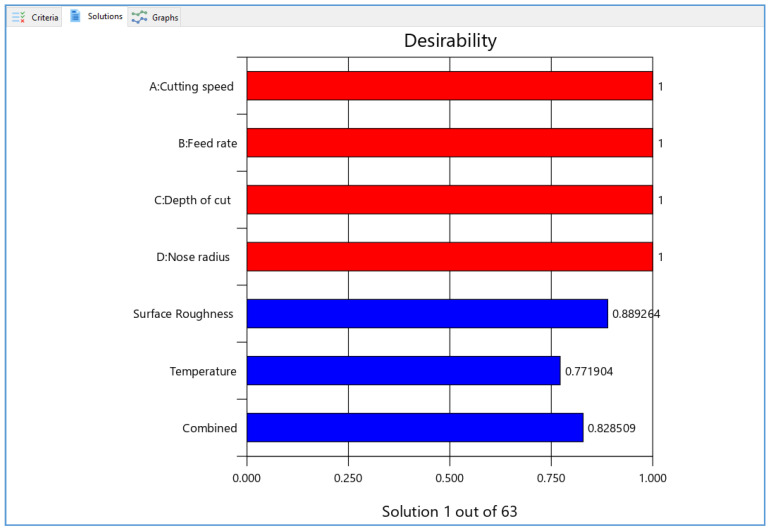
Desirability bar graph for combined optimization.

Figure 7 depicts the ramps of the optimized results. The red dots represent the precise values of the components, while the blue dots represent how well the objectives are satisfied. It is preferable if the ramp is higher up. The relevant desirability bar graph for the provided cutting circumstances, replies, and the combined desirability of 0.829 is shown in Figure 8. The blue bottom bar represents the overall desirability of all the parameters and responses. However, it is essential to note that the objective of an optimization process is to identify an appropriate combination of conditions that meet the goals, not to get a D value of 1. As a result, attractiveness is best accomplished at a low rate of feed and quick speeds while keeping a consistent low cutting depth. Additionally, the ideal solutions and contour map give first-hand data for investigators and industrialists to select optimum machining settings based on design or customer demands. Table 8 shows the optimum values during the turning of aluminum.

## 4. Model Validation

Desirability function analysis is used to estimate the optimal conditions and then validated by physical measurements. The model’s validity is demonstrated using the error percentage of less than ±2%. Surface roughness and temperature testing findings indicate the model is in good agreement with the optimal cutting settings (as predicted by DSA), as shown in Table 9.

## 5. Conclusions

The design of experiment was developed utilizing the central composite design of the RSM to predict surface roughness and temperature rise. Further, desirability function analysis optimization was performed to find the optimum value. The following conclusions have been drawn from this study:

- The cutting speed is the most important influencing parameter compared to the other parameters.
- The surface roughness is minimum at a cutting speed range of 90 m/min to 105 m/min, a rate of feed of 0.09 mm/rev and 0.6 mm and a tool nose radius of 1 mm.
- The temperature is low at a 90 105 m/min cutting speed, and it is much lower when the rate of feed is 0.27 to 0.36 mm/rev. It is observed to be even lower when the nose radius is between 0.6 and 1 mm.
- The desired function optimization strategy is suggested to obtain the optimal tuning parameters. The low surface roughness and temperature value are achievable at a cutting speed of 116.37 m/min, a rate of feed of 0.408 mm/rev, a cutting depth of 0.538 mm, and a tool nose radius of 0.200 mm The related ideal surface roughness and temperature values are 0.374 µm and 27.439°, respectively, with a desirability of 0.829 for the value of 1.

## Figures and Tables

**Table 1:** Chemical properties of aluminum 6061.

Al 6061	Mg	Si	Fe	Mn	Cu	Cr	Zn	Ti	Al
Weight (%)	0.8– 1.2	0.40– 0.80	0.0–0.70	0.15	0.15– 0.40	0.04– 0.35	0.0–0.25	0.0–0.15	Bal

**Table 2:** The factorial levels of output process parameters.

Sl. No	Parameters	Factorial Levels				
		−2	−1	0	1	2
1	Cutting speed, V_c_ (m/min)	75.0	90.0	105.0	120.0	135.0
2	Rate of feed, F_z_ (mm/rev)	0.09	0.18	0.27	0.36	0.45
3	Cutting depth, D_c_ (mm)	0.20	0.40	0.60	0.80	1.00
4	Tool nose radius, R_n_ (mm)	0.20	0.40	0.60	0.80	1.00

**Table 3:** Responses to experimental values.

Sl. No	Cut. Speed (V_c_)	Rate of Feed (F_z_)	Cut. Depth (D_c_)	Tool Nose Radius (R_n_)	Rough. (µm_ob_) (Practical)	Rough. (µm _RSM_) (Pred Using RSM)	Temp. (°C_ob_) Tob (Practical)	Temp. (°C_RSM_) (Pred Using RSM)
1	120	0.36	0.8	0.4	0.38	0.3721	28.8	28.77
2	90	0.18	0.8	0.4	0.44	0.4471	29.8	29.84
3	105	0.27	1.0	0.6	0.41	0.4117	26.6	26.55
4	105	0.27	0.6	0.6	0.39	0.3983	25.8	25.77
5	90	0.36	0.8	0.8	0.46	0.4588	24.9	24.76
6	105	0.27	0.6	0.2	0.41	0.4100	26.9	26.80
7	120	0.36	0.8	0.8	0.37	0.3779	29.9	30.10
8	105	0.27	0.6	0.6	0.39	0.3983	26.2	25.77
9	75	0.27	0.6	0.6	0.49	0.4867	29.5	29.58
10	90	0.18	0.4	0.4	0.43	0.4263	26.8	26.71
11	90	0.36	0.8	0.4	0.45	0.4479	28.6	28.61
12	105	0.27	0.6	0.6	0.42	0.3983	25.6	25.77
13	105	0.27	0.2	0.6	0.39	0.3850	29.6	29.66
14	120	0.18	0.8	0.8	0.40	0.3971	29.8	29.73
15	120	0.18	0.4	0.4	0.39	0.3904	30.1	30.12
16	105	0.27	0.6	0.6	0.39	0.3983	25.6	25.77
17	90	0.36	0.4	0.8	0.45	0.4529	27.8	27.88
18	90	0.36	0.4	0.4	0.44	0.4421	27.2	27.16
19	105	0.27	0.6	1.0	0.41	0.4067	27.1	27.11
20	120	0.18	0.4	0.8	0.37	0.3763	34.2	34.30
21	90	0.18	0.4	0.8	0.41	0.4171	25.8	25.71
22	105	0.45	0.6	0.6	0.41	0.4117	27.6	27.61
23	105	0.09	0.6	0.6	0.42	0.4150	26.8	26.80
24	120	0.36	0.4	0.4	0.36	0.3663	30.4	30.45
25	105	0.27	0.6	0.6	0.39	0.3983	25.8	25.77
26	120	0.18	0.8	0.4	0.41	0.4112	30.1	30.13
27	135	0.27	0.6	0.6	0.37	0.3700	38.4	38.33
28	90	0.18	0.8	0.8	0.44	0.4379	24.2	24.26
29	120	0.36	0.4	0.8	0.38	0.3721	36.5	36.34
30	105	0.27	0.6	0.6	0.41	0.3983	25.6	25.77

**Table 4:** Surface roughness—ANOVA table.

Source	Sum of Squares Value	df	Mean Square Value	F-Value	*p*-Value	
Model	0.0256	14	0.0018	19.96	<0.0001	Significant
V_c_	0.0219	1	0.0219	239.28	<0.0001	
F_z_	0.0000	1	0.0000	0.1745	0.6820	
D_c_	0.0010	1	0.0010	11.17	0.0045	
R_n_	9.000 × 10^−6^	1	9.000 × 10^−6^	0.0982	0.7583	
V_c_ F_z_	0.0016	1	0.0016	17.45	0.0008	
V_c_ D_c_	0.0000	1	0.0000	0.0000	1.0000	
V_c_ R_n_	0.0000	1	0.0000	0.2727	0.6091	
F_z_ D_c_	0.0002	1	0.0002	2.45	0.1380	
F_z_ R_n_	0.0004	1	0.0004	4.36	0.0542	
D_c_ R_n_	0.0000	1	0.0000	0.0000	1.0000	
V_c_^2^	0.0015	1	0.0015	16.83	0.0009	
F_z_^2^	0.0004	1	0.0004	4.21	0.0581	
D_c_^2^	0.0000	1	0.0000	0.0000	1.0000	
R_n_^2^	0.0002	1	0.0002	1.87	0.1916	
Residual	0.0014	15	0.0001			
Lack of Fit	0.0005	10	0.0000	0.2783	0.9596	Not Significant
Pure Error	0.0009	5	0.0002			
Cor Total	0.0270	29				

**Table 5:** ANOVA table of temperature.

Source	Sum of Squares Value	df	Mean Square Value	F-Value	*p*-Value	
Model	311.21	14	22.23	786.09	<0.0001	Significant
V_c_	49.56	1	49.56	1752.53	<0.0001	
F_z_	1.01	1	1.01	35.72	<0.0001	
D_c_	9.70	1	9.70	343.14	<0.0001	
R_n_	0.4290	1	0.4290	15.17	0.0014	
V_c_ F_z_	0.0156	1	0.0156	0.5526	0.4688	
V_c_ D_c_	9.77	1	9.77	345.35	<0.0001	
V_c_ R_n_	26.78	1	26.78	947.06	<0.0001	
F_z_ D_c_	2.81	1	2.81	99.22	<0.0001	
F_z_ R_n_	2.98	1	2.98	105.23	<0.0001	
D_c_ R_n_	20.93	1	20.93	740.18	<0.0001	
V_c_^2^	114.92	1	114.92	4063.88	<0.0001	
F_z_^2^	3.54	1	3.54	125.27	<0.0001	
D_c_^2^	9.37	1	9.37	331.24	<0.0001	
R_n_^2^	2.42	1	2.42	85.49	<0.0001	
Residual	0.4242	15	0.0283			
Lack of Fit	0.1508	10	0.0151	0.2759	0.9606	not significant
Pure Error	0.2733	5	0.0547			
Cor Total	311.63	29				

**Table 6:** Range of parameters and responses for desirability.

Sl.No	Input Parameter	Goal	Lower Limit	Upper Limit
1	V_c_	In range	75	130
2	F_z_	In range	0.09	0.45
3	D_c_	In range	0.2	1
4	R_n_	In range	0.2	1
5	Surface roughness observed (µm)	Minimize	0.32	0.49
6	Temperature rise observed (T_ob_) Degree	Minimize	24.2	38.8

**Table 7:** Best global solution for an optimization.

Number	V_c_	F_z_	D_c_	R_n_	Surface Roughness	Temperature	Desirability	
1	116.37	0.41	0.54	0.20	0.38	27.44	0.829	Selected
2	116.35	0.41	0.54	0.20	0.38	27.43	0.829	
3	116.37	0.41	0.54	0.20	0.37	27.43	0.829	
4	116.34	0.41	0.53	0.20	0.38	27.43	0.829	
5	116.28	0.41	0.54	0.20	0.38	27.43	0.828	

**Table 8:** Optimum values during turning of aluminum.

Sl.No	Input Parameter	Goal	Optimum Value
1	V_c_ (m/mm)	In range	116.371
2	F_z_ (mm/rev)	In range	0.408
3	D_c_ (mm)	In range	0.538
4	R_n_ (mm)	In range	0.200
5	Surface roughness (µm)	Minimize	0.374
6	Temperature rise Observed (T_ob_) (°C)	Minimize	27.439
7	Overall Desirability		0.829

**Table 9:** Optimized process parameter—validation model.

Sl. No	V_c_	F_z_	D_c_	R_n_	Observations	Confirmatory Test		% Error
						Opt. Value by DFA	Exper. Value	
1	116.37	0.41	0.54	0.20	Surface roughness (µm)	0.374	0.370	01.06
					Temperature (°C)	27.439	27.431	00.02
2	114.57	0.38	0.49	0.200	Surface roughness (µm)	0.379	0.373	01.58
					Temperature (°C)	27.406	27.398	00.03

## Data Availability

The data that support the findings of this study are available upon request from the authors.
